# A Praiseworthy Investigation

**Published:** 1921-03-19

**Authors:** 


### TUBERCULOSIS, AGE, AND LOCALITY.

### A Praiseworthy Investigation.

Medical science shares with much else the famous "tendency from the homogeneous to the heterogeneous"; in other words, it is continually becoming more complex and intensive. Now, of course, tuberculosis, especially the pulmonary form, is a most important subject in medical science, while time and space are naturally two great primary relations in which it has to be studied. Yet quite recently, in the case of this country at all events, a clear advance has been made in our knowledge of this prominent disease as regards these two cardinal points. Dr. Brownlee examined the age-incidence of consumption in various localities, and, as might have been predicted, he found not uniformity, but, on the contrary, interesting geographical diversities. In some places there would be an undue proportion of consumption fatal in childhood; in others, at the prime of life; in others, again, at old age—a result, one may repeat, of much interest and promise. The statistics he analysed were from the greater part of the kingdom, and obviously, since a limit to the inquiry had to be drawn somewhere, certain more or less composite, more or less complex, areas had to be treated as simple units. It is therefore now open to keen epidemiologists all over the country to carry on the work still further, contrasting one part of a given area with another almost *ad infinitum* (due regard being paid to the fallacy of small totals), and deducing from the results obtained any legitimate inferences. This task—as regards the second or third most populous sanitary administrative area in England—has just been carried out by Dr. J. B. McDougall, tuberculosis officer to the West Riding of Yorkshire, and his investigation is what the French would call *sensée et sérieuse*. He finds that previous local epidemiological work on phthisis in this part of Yorkshire, although making interesting comparisons of the three Ridings, did not differentiate between various districts of the largest one, which, from many points of view, natural and human—geological, climatic, social, and occupational, for instance—is a highly composite region. Dr. Brownlee himself had lumped it among districts characterised by excess of the "middle-aged" type of phthisis. For further analysis, then, the Riding was divided into the ten administrative areas already adopted for the purposes of anti-tuberculosis work. For each of these ten sub-areas four age groups were selected—fifteen, fifteen to twenty-five, twenty-five to forty-five, and forty-five—and the phthisis death-rates in each group noted. Then from the last census report were obtained the local populations at each age-group. From these two sets of data were calculated a death-rate per thousand. This is a clear and workmanlike beginning, and to see the job in hand well staked out to begin with has pleased critics ever since, at any rate, Goethe's time. Another agreeable feature is the willingness shown to take trouble; for the successful doer, as has been inimitably said, "must be at the beck of the thing to be done."

### The Chief Finding.

We pass to the finding of most prominent interest. Contrast of two north-westerly areas (near Halifax) with others in the extreme south of the Riding—namely, Rotherham, Mexborough, and Swinton—showed that in the latter the juvenile death-rate from pulmonary tuberculosis was much higher, and conversely the "old-age" phthisical mortality distinctly lower. Corroboratively the same phenomenon emerged when figures were examined from county boroughs (which, of course, although geographically within the Riding, are independent administratively) in the neighbourhood of these contrasted districts respectively. The same difference again, held good when the criterion taken was not consumption, but non-tuberculous respiratory diseases, such as influenza, pneumonia, bronchitis, and broncho-pneumonia. Of this south part the worst spot for juvenile phthisis was found to be Bolton-on-Dearne (parenthetically we are glad to say that this municipality has, since the war, taken a praiseworthy lead in the matter of putting up working-class houses). Thus geographically there appears to be distinctly more juvenile consumption and distinctly less consumption of elderly persons in the south of the West Riding of Yorkshire than in its north-western portion—and Dr. McDougall, as tuberculosis officer of the region, is naturally intrigued to get at the cause of this.

### Capturing the Critic.

Here, perhaps, he must forgive us if we part company from him a little: authors rarely succeed in capturing the critic body and bones. What observant person does not, at heart at any rate, agree with Mr. Hilaire Belloc in his stinging political satires? But the number of those who subscribe to his remedies for the abuses he exposes is vastly less. However, again Dr. McDougall begins well. He gets for us the fact that these same southerly districts that show excess of juvenile pulmonary infection also show excess of infantile mortality. Next, as to occupation, these southerly districts are clearly marked off as the chief colliery districts in the Riding. Now colliers, as a class, are but little subject to phthisis. He is able easily to exclude geological differences as invokable causes of the heavy toll on child life, except in some slight measure where surface subsidence due to mining underneath leads to waterlogging. Climate is shown to have a certain small determining influence. Housing accommodation is bad everywhere, and sometimes at its worst and scantiest where phthisis is not particularly rife. This is a finding that needs emphasising, for the panacea of better housing as a certain anti-tuberculosis measure, instead of general social amelioration, has been advanced again and again. After a full discussion of possible

**Tuberculosis, Age, and Locality**—(*continued*).

etiological factors, he concludes, *inter alia*, that there is probably a certain amount of immunity to pulmonary disease amongst adults in the southern area named, and that the factor which decides a high infantile and juvenile mortality rate is apparently atmospheric contamination, most common in mining and industrial centres with a low rainfall and situated at a low altitude.

As to the immunity, we agree. But it is probably (apart from the occupational advantage above mentioned) a natural, not an acquired one. Some years ago there was a proposal in *Biometrika* to study in a district high juvenile death-rates, and also adult death-rates at a subsequent period, it being argued that likely enough the latter would be found to be low, since all the weakly persons would have been killed off early. We fancy the statistician who suggested this particular *enquête* would be glad of Dr. McDougall's tuberculosis and respiratory death-rates in these southern districts as verifying such a theory. Moreover, as regards tuberculosis, it should be recalled that when the suggestion was first made not to sterilise milk for children, but to let the bovine tubercle bacilli therein act as a vaccine against the human form in later life, a shrewd Continental criticism on the proposal was that individual tuberculosis immunity is a very frail and evanescent phenomenon, and that the tendency to relapse and re-activation of any tuberculous lesion was a drawback more than cancelling the advantage from it. As to atmospheric contamination, that is, of course, a factor that has been mentioned lately with regard to the question of infantile mortality generally. But is a mining district any murkier than a manufacturing one? We doubt it. And has not consumption been successfully treated on Whitechapel balconies? The real clue may perhaps be found to be bound up with occupation. Miners' children have a higher mortality rate than those of almost any other class, for some reason—perhaps partly from the domestic upset working on night shifts makes, and consequent neglect. But to the author saturated with his subject such comments may seem irritating. We notice this inquiry at length because, done in this thorough fashion, it is the sort of investigation that ought already to be in hand in every large sanitary area.

